# Constitutional Origin of Diphtheria

**Published:** 1880-06

**Authors:** R. M. Griswold

**Affiliations:** North Manchester, Ct.


ARTICLE II.

### CONSTITUTIONAL ORIGIN OF DIPHTHERIA.

BY R. M. GRISWOLD, M. D., NORTH MANCHESTER, CT.

Were it not for its influence upon the treatment of diphtheria the question of constitutional or local origin of the disease would be of comparatively little practical benefit to the profession; but when we consider that upon its proper and intelligent treatment depend the lives of many of our little patients, (and of some of our older ones,) the question becomes one of vital importance.

No question has so agitated the mind of the medical fraternity in this country for the past four or five years, as this one. Instances and authorities, cases in practice and experiments, have been quoted, pro and con, without number, and the medical mind is still divided. I shall attempt in this article to bring some points to bear, tending to show that diphtheria is a disease of constitutional origin, and that the local manifestations are but secondary to the constitutional symptoms.

While giving all due deference to the illustrious men who have investigated by experiment, this disease, and who originated and so ably maintain the theory of local origin, yet we must remember that "it is a lamentable fact that great authorities are not *always* right," and that in this matter, as in every other disease which we attempt to study, *facts* are one thing and *theory* is another, and very often the facts and the theory do not agree.

"Diphtheria is as much a disease of the general system as scarlet fever or small pox, in the latter the especial place of localization is usually the outer coverings, in the former the mucous membranes. I

am certain that I have seen cases where the diphtheritic disease was dominant, when all symptoms were manifest, but where there was no development of exudation. Such cases have their counterpart in all epidemics of measles. It is not uncommon to see a case, among three or four in a family, when the other symptoms are severe, but where there will be no exception, meanwhile the other cases, dating from the same point of exposure, developing the premonitory symptoms at the same time, and proceeding in other respects like the non-eruptive case, will have a free skin development. The non-eruptive case is none the less a 'general' or 'blood poisoned' one, nor in many instances is the system any the less under the influence of the specific virus. In fact the non-eruptive cases are the most dangerous, as the poison is laying its force upon the mucous membranes, and instituting pulmonary or intestinal irritation. The deduction from this, in its analogous application to diphtheria is, that we may, and frequently do have, cases in which there is no membranous deposit at all. In respect to the deposit the case *aborts*. Upon inspection of the fauces in these cases, they will present an appearance unlike that in any other kind of sore throat; a shining, glassy look, an indication of an attempt to lift the mucous membranes from the tissues beneath, and in some instances a distinct line of demarcation, between an arterial and venous coloring in the membrane itself, sufficient to show an attempt at exudation. More than this, before *any* local manifestations can be discovered, there is frequently a general depression of the whole system; a severe chill followed by high fever, and extreme prostration."

Now, if diphtheria is "at first a local disease," and "general disease" is dependent upon the progress of the local trouble, we may ask, why this general disturbance before any local manifestation is present?* Is it not as reasonable to suppose that in small pox the fever is dependent upon the eruption, or that in measles we should have the eruption inducing the fever? Common sense alone should teach us that these are but local manifestations of a constitutional disease, as the intestinal lesions are local manifestations of typhoid, or as chills, fever and sweating are local manifestations of malaria.

But it may be said that diphtheria can be positively demonstrated only by the presence of the peculiar exudation, to which we reply, that the cases of any febrile disease are not *all* typical in possessing every one of the symptoms which belong to that peculiar disorder.

*Griswold Proceedings, Conn. St. Med. Society, 1878.

In an epidemic of rubeola, we have cases with all the other symptoms pronounced, but no rash; cases with the eruption, but no fever, and no catarrhal trouble, and with catarrhal trouble and fever but no rash; cases with the eruption and fever, and the fully developed disorder. You may call them *spurious* cases, but we reply, they are all measles, and all alike owe their origin to the same specific contagious influences. There is only a diversity in the development of symptoms. Corresponding observations may be made upon the phenomena presented by diphtheria. There will be variety in the symptoms, manifestly, the membranous deposit may be wanting to give the fully developed typical case, but other indications will assure you that the diphtheritic influence is there."*

If the disease is of local origin, how are we to explain those cases terminating fatally in from twenty-four to forty-eight hours? Is it possible for a local disease to terminate thus suddenly, the life of a patient? And yet, nearly every one who has had any extended experience with this disease, has seen cases terminating fatally in this short time, that were without the slightest doubt, diphtheria. In the spring of 1875 I was called to see a child who had been in apparent health twenty-four hours previous; I found him with very high fever exceeding rapid and feeble pulse, and extreme general prostration. In twenty hours after I saw him he was dead. The third day after, a sister, a year or two older, manifested the same symptoms, and died in less than forty-eight hours. In neither case was there the slightest indication of any local diphtheritic trouble, and I was somewhat at a loss for a diagnosis. Within six days two other members of the family were prostrated, and with all the other usual symptoms, presented the typical exudation of diphtheria, confirming beyond a doubt the opinion that the two fatal cases were of the same nature, but that the general constitutional disturbance was so severe that they died before any local affection presented.

Upon this point, Dr. George Johnson, in the *Lancet*, Jan. 16th, 1876, says, "in most cases of diphtheria, there is abundant evidence of blood infection during the progress of the malady, the high temperature, the general constitutional disturbance and the nervous symptoms, and sequels are results probably of blood poisoning. *There is a class of cases in which constitutional symptoms, the result of blood poisoning, are overwhelming and rapidly fatal, while there is little or no appearance of membrane upon the throat.*"

*(Griswold, in Proceedings of Conn. Medical Society, 1876.)

The doctrine of local origin is supported by many upon the ground of evidence adducted from cutaneous diphtheria; that under certain favoring circumstances a diphtheritic exudation may make its appearance upon abraded surfaces, is true, but let us for a moment consider this fact.

1st. Cases of cutaneous diphtheria are rare, and it will be found in the great majority of cases, that it occurs in those persons who have been for some time under the influence of the peculiar diptheritic virus, and that more frequently still it occurs as secondary to the pharangeal disease.

2d. Other cases of cutaneous diphtheria are traceable directly to contact of secretions from diphtheritic patients, with abraded surfaces. The following case will illustrate this:

*Case 1.* In October, 1878, I saw a child three years of age, which had diphtheria. The local throat manifestations were quite prominent. After weaning, it had acquired the habit of sucking its thumb, and while endeavoring to break it of this, the mother occasionally gave it one of her fingers to suck; upon the third day, the mother showed me a scratch upon her finger which was covered with an unmistakable diphtheritic exudation. It pained her considerably, and was much inflamed. I applied a solution of argenti, nit. and in two days the exudation disappeared. There was no constitutional disturbance.

*Case 2.* This case was mentioned by the late Dr. George A. Moody, of Plainsville, Conn., at the meeting of the Hartford County Medical Society, April 27th, 1875, and is quoted by Dr. J. W. Lyon, of Hartford, in a paper upon diphtheria, read before the State Society the following year.

"A child was seized with diphtheria of the fauces, February 3d, 1875, and died upon the 17th. A lady who had an abrasion upon her thumb, attended upon it the 16th and 17th, and after its death washed the body and laid it out. There was a discharge from the nose which she frequently wiped away, and it is certain that some of this matter came in contact with the sore upon her thumb. She came home upon the evening of the 18th, and her thumb was so painful that she poulticed it without alleviation. In the morning I saw her and found her thumb covered with an exudation resembling diphtheria. I applied the dry pursulphate of iron, which soon changed the character of the sore, and it healed rapidly. There was some constitutional disturbance, headache and fever, which subsided with the improvement of the sore."

That constitution symptoms and systemic infection may follow cutaneous diphtheria, cannot be denied, and while we admit the possibility of cutaneous diphtheritic exudations being formed by atmospheric influences, we must consider it as only theoretical, while we KNOW that cutaneous diphtheria may be, and is produced by actual contact with diphtheritic secretions.

Dr. Irving W. Lyon, of Hartford, Conn., in an article upon diphtheria, published in 1876, enumerates the following conclusive reasons as evidence of the constitutional or blood origin of this disease.

1st. It occurs in epidemics.

2d. These epidemics are variable in type, as in other acute infectious diseases.

3d. It has a period of incubation ranging from two to fourteen days.

4th. One or more of the following constitutional symptoms, viz.: malaria, drowsiness, chilliness, occasionally amounting to shivering, febrile reaction with headache, aching of the limbs and loins, and sometimes nausea and vomiting, may precede the faucial exudation from a few hours to a few days.

5th. That the disease is communicated to others by contagion, and by inoculation.

6th. That adynamic and toxic symptoms more or less profound, rapidly follow the invasion of the disease.

7th. In the rapidly fatal, malignant form, there seems to be a simultaneous poisoning of the whole system. When the characteristic pellicle BEGINS to appear on the tonsils and in the nasal passage, the whole passage economy is ALREADY profoundly altered.

8th. The occurrence of paralysis as a sequal to a certain proportion of cases."

We quote the following from the reply of Dr. Rufus W. Griswold, of Rocky Hill, Ct., President of the Hartford County Medical Society, to the circular of the committee, on matters of professional interest in the State, as showing the period which may intervene between the invasion of the disease and any local manifestations.

"Mrs. G. of Wethersfield, was at my residence on the evening of February 15th, 1877; had been feeling unwell through the day.

Attended a meeting that evening, had severe rigors while there; returned to my house, and after trying to get warm, went home. Had high fever come on during the night, with severe pains in different parts of the body, especially the arms. I found her in this condition next day. No sore throat, no indications of diphtheria. The season and some exposure made pneumonia much more probable. The next day the disease localized itself in the throat, and developed very slight exudation. The constitutional disturbance in this case was severe, and preceded any appreciable localization in the throat by forty-eight hours. And this is the history of a great many cases. It argues a constitutional diphtheritic poisoning, preceeding any local trouble."

From the conclusions, we can draw but one inference; if we give weight to *facts*, rather than *theory*, viz.: that diphtheria is primarily a constitutional disease, which usually selects the throat as the centre of localization, because it is that mucous membrane which is most susceptible, and that only by inoculation can we produce a local exudation, without a previous constitution disturbance. Two other points which strongly indicate diphtheria to be a constitutional disease, are embodied in the report of a number of eminent physicians to the Legislative Assembly in Victoria, in 1872.

1st. Diphtheria may occur in *any* locality, and *under every condition.* Now, if diphtheria begins as a local disease, the result of certain parasitic lodgements in the larynx and trachea, certain conditions favorable to the development of those parasites, must seem to be necessary. A certain unascertained degree of heat or cold, of dryness or moisture. But we are told that it may occur in any locality, and *under every condition.* We must admit that parasitic growths are developed in the course of most diphtheria, but they are developed as maggots are developed in a sore, they are the outcome of the disease, which under certain favoring atmospheric conditions, and certain favoring conditions of the system, which have never been, and probably never will be explained satisfactorily, are developed.

2d. "Recurrence in the same individual is not the rule, and the recurrent causes are less severe than the original attack."

I think most physicians in this country will coincide with me in saying that this statement is correct; and that the occurrence of a second attack of diphtheria in the same person is the exception and not the rule.

I know of no *local* disease that is not as apt to occur in a person a second time as the first, but in the majority of constitutional diseases one occurrence in the same person is the rule. We must admit therefore that diphtheria is an exception to other diseases, in that it is not apt to occur twice in the same individual, or we must admit that it is of constitutional origin.

In conclusion, we can only say that we have attempted to study the origin of diphtheria from a common sense view of the disease, not from a theoretical one; perhaps therefore, not from a scientific one, but from a view of the case as it is presented us in ordinary practice I am sure that in this section, and I think it is the same in other sections of country; those physicians who treat the disease upon constitutional principles, regarding the local manifestations only as secondary, and removing the exudations only as we would remove any accumulation or growth from a sore, only when it interferes with the comfort or safety of the patient, are those who are meeting with the greatest success, and success in the saving of our little patients is what we are after.

